# Three-Dimensional Infinite Cluster Function as a Descriptor of Through-Plane Effective Conductivity in Porous Electrodes of Membrane Electrode Assemblies

**DOI:** 10.3390/ma19050835

**Published:** 2026-02-24

**Authors:** Abimael Rodriguez, Jaime Ortegón, Abraham Rios, Carlos Couder, Romeli Barbosa

**Affiliations:** 1 Division of Sciences and Engineering, Universidad de Quintana Roo, Boulevard Bahía s/n, Chetumal 77019, Mexico; catedra.abimael@uqroo.edu.mx (A.R.); jortegon@uqroo.edu.mx (J.O.); abraham.rios@uqroo.edu.mx (A.R.); 2 Instituto Politécnico Nacional, Aerospace Development Center, Belisario Domínguez 22, Col. Centro, Del. Cuauhtémoc, Mexico City 06010, Mexico; 3 Renewable Energy Unit, Centro de Investigación Científica de Yucatán, C 43 No 130, Chuburná de Hidalgo, Mérida 97200, Mexico

**Keywords:** 3D microstructure reconstruction, effective conductivity, infinite cluster function, microstructural connectivity, percolation theory, finite-volume simulation, structure–transport relationships, membrane electrode assembly, PEMFC electrodes

## Abstract

Through-plane electronic transport in porous membrane electrode assembly (MEA) electrodes is governed by the three-dimensional (3D) connectivity of the conducting phase. Here, we quantify the role of the spanning-cluster fraction $P_{\infty }$, defined as the fraction of conducting-phase voxels that belong to the z-spanning connected component in a finite reconstructed volume, on effective conductivity using scanning electron microscopy (SEM)-informed 3D reconstructions of four archetypal morphologies: a granular catalyst layer (CL), labeled CL1; a fibrous gas diffusion layer (GDL), labeled GDL1; an open-cell foam (OCF); and a micro-fibrous non-woven (MFM), labeled MFM1. Each morphology is reconstructed on a 150×150×150 voxel grid, and z-spanning connectivity is identified with a 26-neighbor flood-fill algorithm. Steady-state conduction is solved by a finite-volume method (FVM) with an imposed potential difference between the *z*-faces and no-flux lateral boundaries. Although all samples exhibit through-thickness connectivity, the normalized conductivity $\sigma_{eff}/\sigma_{bulk}$ varies widely, from ≈0.134 (MFM1) to ≈0.706 (OCF). The corresponding ${(P}_{\infty },\sigma_{eff}/\sigma_{bulk})$ pairs are $\left(0.996,\approx 0.306\right)$ for CL1, $\left(0.999,\approx 0.303\right)$ for GDL1, $\left(0.997,\approx 0.706\right)$ for OCF, and $\left(0.901,\approx 0.134\right)$ for MFM1. OCF exhibits the highest response due to vertically coherent channels, whereas MFM1 underperforms due to laminated constrictions; CL1 and GDL1 lie in an intermediate regime with nearly isotropic skeletons. Overall, the results show that while a z-spanning connected component is required for measurable conduction, the magnitude of $\sigma_{eff}$ is dictated by percolating-skeleton quality (bottlenecks, cross-sectional constrictions, and pathway alignment) rather than phase amount alone. The proposed descriptors therefore enable percolation-aware screening metrics for designing and comparing MEA-relevant GDL and CL microstructures.

## 1. Introduction

The performance of membrane electrode assemblies (MEAs) in proton exchange membrane fuel cells (PEMFCs) is strongly governed by the microstructural characteristics of their porous electrodes, namely the gas diffusion layer (GDL) and the catalyst layer (CL). In MEAs, the proton exchange membrane (PEM) provides ionic conduction between electrodes; perfluorosulfonic acid (PFSA) membranes remain the most widely used commercial PEM technology [1].

Under operation, radical-driven chemical attack can induce polymer degradation, sulfonic group loss, and membrane thinning or pinhole formation, which reduces proton conductivity and contributes to ohmic losses [1,2]. While membrane durability is critical at the device level, the present study focuses on the porous electrodes (GDL/CL) and isolates how their three-dimensional 3D electronic connectivity governs the electrode-side contribution to effective conduction.

These components regulate reactant transport, water management, and charge conduction, all of which depend critically on the degree of connectivity across the microstructure. Accurate modeling of these electrodes is therefore essential for predicting effective transport properties and guiding MEA design. Although two-dimensional (2D) models are widely used due to their simplicity and computational efficiency, they exhibit fundamental limitations when applied to porous electrodes. Several studies have shown that 2D approximations often misrepresent porosity, permeability, and connectivity, as they cannot fully capture the complex 3D structure of pore networks [3,4,5].

These discrepancies are particularly evident in flow and velocity field predictions, where 3D effects such as hydrodynamic dispersion and anisotropy dominate [6]. Furthermore, the concept of representative elementary volume (REV) differs between 2D and 3D systems, introducing additional scale-dependent errors. As a result, 2D models can underestimate or overestimate effective transport coefficients, particularly in heterogeneous porous electrodes where bulk properties are governed by volumetric pathways and percolation [3]. Three-dimensional modeling is therefore indispensable when connectivity, anisotropy, and multiphase interactions significantly influence transport behavior. In the GDL, the anisotropic orientation of carbon fibers cannot be represented in 2D, while in the CL, isotropic granular aggregates require volumetric resolution to realistically capture phase continuity. Transport properties such as permeability and conductivity are often controlled by pathways that extend in all directions, which necessitates volumetric modeling [7,8,9,10,11,12]. Even when 2D-to-3Dreconstruction techniques are employed, full 3D modeling remains essential for validation and for capturing anisotropic effects and multiphysics interactions [13]. A key concept for describing transport in such microstructures is the formation of infinite percolation clusters.

These clusters represent continuous, system-spanning pathways that sustain long-range conduction, in contrast to isolated clusters that do not contribute to macroscopic transport [14,15,16,17]. Within the infinite cluster, transport efficiency depends on the connectivity of its backbone, whereas side branches and dead ends contribute only marginally and can even introduce anomalous effects [18,19]. The onset of effective transport is closely associated with the percolation threshold, beyond which transport coefficients increase sharply following universal scaling laws [20,21]. The geometry and morphology of the infinite cluster directly influence scaling exponents for conductivity and diffusivity [22]. Thus, detecting the formation of an infinite cluster provides a robust criterion for determining whether a porous electrode can sustain macroscopic transport. Recent advances in microstructural reconstruction have enabled the generation of statistically accurate 3D volumes from limited 2D image data. High-resolution experimental methods such as X-ray computed tomography and focused ion beam–scanning electron microscopy (FIB-SEM), along with computational approaches including generative adversarial networks (GANs), diffusion models, and recurrent neural networks, have demonstrated strong capabilities for reproducing complex porous media with high fidelity [9,23,24,25,26]. However, these techniques often require large datasets and computational resources. Optimization-based strategies, such as simulated annealing, remain relevant when training data are limited, as they iteratively adjust structures to match statistical descriptors like the two-point correlation function $S_{2}$ or the lineal-path function $L_{p}$ [6,27]. Hybrid hierarchical strategies have further enhanced convergence and representational power, making simulated annealing a practical option for applications that prioritize geometric and statistical consistency. Despite these advances, most statistical descriptors capture morphology but not long-range connectivity. For instance, $S_{2}$ reflects phase distribution but is insufficient to describe percolation, while $L_{p}$ provides directional continuity but lacks global information. In this work, we therefore introduce the infinite cluster function $P_{\infty }$, defined as the fraction of phase voxels belonging to the system-spanning cluster, as a compact descriptor of long-range connectivity in MEA electrodes [28]. Fuel cell MEAs translate microstructure into three dominant performance channels: ohmic losses, oxygen transport, and water management. Accordingly, we connect $P_{\infty }$ and a small set of backbone-level metrics ($f_{bb},B,\tau_{z},A_{z}$) to MEA-relevant proxies such as electronic and protonic area-specific resistance (ASR) and oxygen-transport resistance [29,30,31].

To provide a quantitative bridge to PEMFC performance, we interpret the computed through-plane effective conductivity $\sigma_{eff,zz}$ in terms of an electrode-side electronic area-specific resistance scaling. At the layer level, the electronic ASR contribution scales as $ASR_{e}∼L_{z}/\sigma_{eff,zz}$. To reflect that long-range conduction in finite volumes is sustained only by the spanning fraction of the solid phase, we also introduce a connectivity-weighted indicator $ASR_{e}^{*}∼L_{z}/(\sigma_{eff,zz}P_{\infty })$, used here as a robustness/design proxy rather than a strict constitutive law. Supplementary Table S1 [32,33,34,35] benchmarks the order of magnitude of bulk and interfacial resistances reported for diffusion-media assemblies and motivates the relevance of both connectivity ($P_{\infty }$) and skeleton quality (captured by $\sigma_{eff}$ and backbone metrics). Limiting-current behavior is primarily governed by oxygen transport through the pore network at high current density and is therefore outside the scope of the present electronic-network analysis. This framing enables structure-informed design rules at the layer level (GDL, microporous layer and CL) and across interfaces, highlights the infinite cluster function and associated connectivity descriptors as complementary criteria for connectivity and transport prediction in MEA electrodes. While idealized template microstructures (e.g., periodic lattices, zeolite-like networks, or ideal scaffolds) are useful when the research question targets a prescribed architecture, substantial evidence shows that such templates can misrepresent scale-dependent connectivity and may bias transport predictions (often overestimating effective permeability/diffusivity)unless carefully calibrated against image-based or experimental microstructures and accompanied by explicit REV analysis [32]. Therefore, rather than adopting a single canonical geometry, we use an architecture-agnostic percolation framework to compare distinct morphology families on a consistent connectivity basis [33,34]. Because effective transport depends on 3D connectivity and pathway quality (e.g., constrictivity, tortuosity, and anisotropy), predictive modeling is required to link real microstructure to effective properties beyond qualitative morphology inspection. Virtual materials testing has demonstrated that quantitative microstructure–property relations enable robust comparison and ranking across morphology families [32,35]. This study builds upon our earlier two-dimensional 2D analysis of synthetic agglomerate structures, which showed that specific geometries can promote percolation and enhance effective electrical conductivity even at low surface fractions [30]. However, that framework was restricted to idealized 2D domains and could not assess through-plane connectivity in realistic MEA architectures. Here, we extend the approach to three dimensions by evaluating the infinite cluster fraction $P_{\infty }$ on SEM-informed 3D reconstructions of four archetypal morphologies, a granular catalyst layer, a fibrous gas diffusion layer, an open-cell foam, and a micro-fibrous non-woven support, and by coupling $P_{\infty }$ to finite-volume simulations of steady-state conduction to compute the normalized effective conductivity σ_eff_/σ_bulk_. We show that $P_{\infty }$ provides a practical 3D measure of through-plane connectivity and that its joint analysis with σ_eff_/σ_bulk_ disentangles (i) the onset of a z-spanning cluster from (ii) the transport-limiting quality of the percolating skeleton, as captured by backbone continuity, bottleneck severity, through-plane tortuosity, and path alignment. Overall, this work contributes a percolation-aware 3D workflow that separates connectivity existence (via $P_{\infty }$) from pathway quality (e.g., B, τ_z_, $A_{z}$, and $f_{bb}$) to explain and ultimately optimize through-plane effective conductivity across MEA-relevant morphologies, enabling consistent comparisons across architectures and actionable microstructure-level design guidelines for GDLs and CLs in PEM-based energy devices.

## 2. Microstructural Characterization and Image-Based Methodology

**Figure 1:**
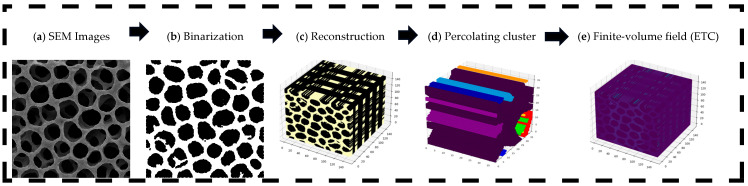
Methodological pipeline for connectivity and transport analysis in porous membrane electrode assembly (MEA) electrodes. (**a**) scanning electron microscopy (SEM) images; (**b**) binarized masks yielding solid/pore phases; (**c**) three-dimensional (3D) statistical reconstruction consistent with prescribed descriptors; (**d**) connectivity map highlighting the percolating (system-spanning) cluster retained for simulation; (**e**) representative finite-volume solution field used to compute effective properties.

This section describes the image-based workflow used to analyze connectivity and transport in porous electrodes of membrane electrode assemblies (MEAs). The framework integrates experimental imaging (e.g., SEM), digital image processing, three-dimensional statistical reconstruction, connectivity analysis, and finite-volume transport simulations. By combining statistical descriptors with numerical modeling, the workflow bridges microstructural features with effective transport properties. The overall pipeline is summarized in Figure 1 and proceeds through the following stages.

### 2.1. SEM Imaging of Porous Electrodes

Microstructures of the gas diffusion layer (GDL) and the catalyst layer (CL) were obtained from representative SEM images of commercial or prototypical MEA electrodes. In addition to these two electrode types, two further architectures were considered as archetypal morphologies for comparison: an open-cell foam and a micro-fibrous non-woven support. The selected micrographs capture the characteristic granular texture of CLs, the fibrous architecture of GDLs and the more open, reticulated structures of foam and non-woven supports. All images were converted to grayscale and standardized in terms of spatial resolution and intensity range prior to segmentation, ensuring a consistent pixel size and contrast across samples. Binarization was carried out in two stages. First, a global Otsu threshold was applied to obtain an initial separation between solid and pore phases. Second, local threshold refinement and mild contrast enhancement were used to correct for uneven illumination and to preserve fine features without saturating bright edges. Post-processing was deliberately minimal and isotropic: small, isolated islands were removed, and single-pixel holes were filled using a small structuring element to avoid directional bias. Quality control was performed visually and via simple statistics to ensure that segmentation neither artificially overconnected nor broken relevant structural features. The resulting binary images, encoded as arrays with solid voxels equal to 1 and pore voxels equal to 0, served as input to the 3D reconstruction stage.

### 2.2. 3D Statistical Reconstruction

The statistical similarity between the reconstructed and target microstructures was evaluated using three correlation-based descriptors: the two-point correlation function $S_{2}\left(r\right)$, the lineal-path function ($L_{P}\left(r\right)),$ and the pore-size distribution $P_{s}\left(r\right)$. Their mathematical formulations are shown in Equations (1)–(3) and follow the definitions presented in [36].

#### 2.2.1. Two-Point Correlation Function

The two-point correlation function $S_{2}\left(r\right)$ quantifies the probability that two points separated by distance r lie in the same phase:

(1) $$S_{2}\left(r\right)=\langle I\left(x\right)I\left(x+r\right)\rangle ,$$

where $I\left(x\right)$ is the indicator function of the solid phase, taking the value 1 if the point x belongs to the solid and 0 otherwise.

#### 2.2.2. Lineal-Path Function

The lineal-path function $L_{P}\left(r\right)$ measures the probability that a line segment of length r is entirely contained within the same phase:

(2) $$L_{P}\left(r\right)=\langle \prod_{i=0}^{r}I\left(x+ie\right)\rangle$$

where $I\left(x\right)$ is the indicator function of the phase of interest, e is the unit vector defining the orientation of the segment, and the product operator ensures that the line segment contributes only if all points remain within the same phase.

#### 2.2.3. Pore-Size Distribution Function

The pore-size distribution $P_{S}\left(r\right)$ is defined as the probability density of finding the largest sphere of radius r that can be inscribed within the pore phase. It is obtained from the derivative of the pore survival function $F\left(r\right)$:

(3) $$P_{S}\left(r\right)=-\frac{dF\left(r\right)}{dr}$$

where $P_{S}\left(r\right)$ is the pore-size distribution function, representing the probability density of pores of radius r; $F\left(r\right)$ is the pore survival function, which gives the probability that a randomly selected point within the pore space lies at least a distance r away from the pore–solid interface; r is the pore radius, i.e., the radius of the largest inscribed sphere within the pore phase; and $-\frac{dF\left(r\right)}{dr}$ the negative derivative ensures that $P_{S}\left(r\right)$ remains positive, since $F\left(r\right)$ monotonically decreases as r increases. This formulation allows for quantifying the distribution of pore sizes in terms of local geometrical constraints, complementing other statistical descriptors such as the two-point correlation and lineal-path functions [37].

#### 2.2.4. Simulated Annealing (SA) Reconstruction

The simulated annealing (SA) reconstruction follows the statistical framework introduced by Yeong and Torquato [38,39,40,41], where stochastic optimization is used to match target descriptors extracted from 2D microscopy. For image acquisition, scanning electron microscopy (SEM) micrographs were obtained using a scanning electron microscope (JSM-6360LV, JEOL Ltd., Akishima, Tokyo, Japan). Under fixed phase-fraction constraints, SA generates statistically consistent 3D reconstructions under fixed phase-fraction constraints. An initial 3D configuration is first generated to satisfy the prescribed phase fraction. At each SA step, two voxels from opposite phases are randomly selected and their labels are swapped (1 ↔ 0), which preserves the global phase fraction exactly. The reconstruction error is defined as a lag-averaged mean-squared mismatch between the target descriptors and those computed from the current 3D volume over $N_{\mathrm{lags}}=\lfloor L/2\rfloor$ discrete distances (with $L=150\Rightarrow N_{\mathrm{lags}}=75$). In the present implementation, the objective aggregates the squared errors of the two-point correlation function $S_{2}(r)$ and the lineal-path function $L_{p}(r)$ for both phases (solid and pore), using equal weights for all terms:

(4) $$E=\frac{1}{2N_{\mathrm{lags}}}\sum_{r}[{\left(S_{2,\mathrm{solid}}^{\mathrm{rec}}(r)-S_{2,\mathrm{solid}}^{\mathrm{tar}}(r)\right)}^{2}+{\left(L_{p,\mathrm{solid}}^{\mathrm{rec}}(r)-L_{p,\mathrm{solid}}^{\mathrm{tar}}(r)\right)}^{2}+{\left(S_{2,\mathrm{pore}}^{\mathrm{rec}}(r)-S_{2,\mathrm{pore}}^{\mathrm{tar}}(r)\right)}^{2}+(L_{p,\mathrm{pore}}^{\mathrm{rec}}(r)-{L_{p,\mathrm{pore}}^{\mathrm{tar}}(r))}^{2}].$$

For quantitative reporting, we also provide the equivalent global RMSE across all matched targets, ${RMSE}_{global}=\sqrt{E/2}$. For the four representative reconstructions analyzed in this work (OCF, CL1, MFM1, and GDL1), the converged costs were $E=4.41\times 10^{-11}$, $1.32\times 10^{-9}$, $1.19\times 10^{-9}$, and $1.34\times 10^{-9}$, respectively, corresponding to ${RMSE}_{global}=4.70\times 10^{-6}$ and $\left(2.44\;to\;2.59\right)\times 10^{-5}$. These values confirm a very small quantitative mismatch between target and reconstructed descriptor curves beyond visual agreement.

Candidate moves are accepted using the Metropolis rule $P_{accept}=min\{1,exp(-\Delta E/T)\}$, where ΔE is the change in the objective function and T is the current temperature. The temperature follows a geometric cooling schedule $T_{k+1}=\alpha T_{k}$, with α=0.999999999 and $T_{0}=1\times 10^{-6}$. In the implementation, the temperature is updated after each attempted trial move (one trial move per temperature update). The SA loop terminates when $T\leq T_{min}$ and/or when the objective error falls below $\varepsilon =5\times 10^{-10}$. The minimum temperature is defined as $T_{min}=T_{0}(0.1{)}^{20\lceil {log}_{2}(L)\rceil }$. The pseudo-random generator is initialized with a time-based seed in the current implementation; for exact reproducibility, a fixed seed can be used, or the seed can be recorded and reported per run (Table 1).

### 2.3. Connectivity Analysis: Percolation and Infinite Cluster Function

**Figure 2:**
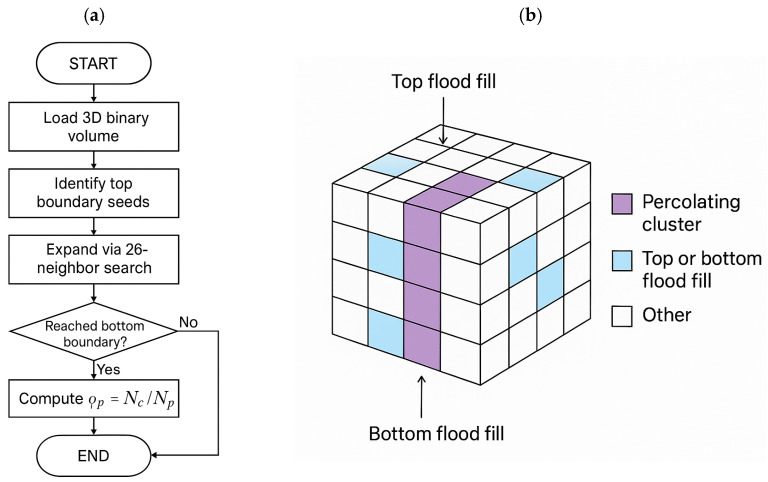
(**a**) Flowchart of the 3D flood-fill algorithm used for spanning-cluster detection in reconstructed porous microstructures. The workflow begins with binary voxel data, identifies phase voxels on the top boundary, and iteratively expands through all connected neighbors using a 26-neighbor criterion until the connected set saturates. (**b**) Three-dimensional schematic representation of the flood-fill process showing the propagation of connectivity through the network from top to bottom. The highlighted voxels represent the z-spanning cluster that establishes a continuous transport path between opposite boundaries.

Connectivity within the reconstructed 3D microstructures was evaluated using a percolation-based approach to identify continuous transport pathways across the phase of interest. In finite voxel domains, percolation is commonly assessed by the existence of a system-spanning connected component that links opposite boundaries of the sample. The presence of such a spanning cluster indicates that a long-range transport pathway exists, enabling effective conduction or diffusion through the porous network [42]. Several approaches exist to identify connectivity in discretized porous systems. Geometric methods such as flood-fill, union–find, or Hoshen–Kopelman labeling algorithms rely on voxel connectivity criteria (typically 6-, 18-, or 26-neighbor definitions) to detect system-spanning clusters. Probabilistic formulations are often used to discuss the percolation threshold $p_{c}$, which describes the minimum phase fraction required for the emergence of spanning connectivity in an ensemble sense [43,44]. Functional methods evaluate connectivity indirectly through physical simulations (finite-volume or finite-element), where the onset of a nonzero effective transport coefficient indicates a connected pathway [45].In this work, we implemented a 3D flood-fill to test through-plane (z) connectivity across the phase of interest (pore for ionic cases, solid for electronic cases). The algorithm starts from voxels on the top face, explores all connected neighbors using a 26-neighbor rule, and stops when no new voxels can be reached. If the visited set touches the bottom face, a z-spanning cluster is identified. This method is linear in the number of voxels, directionally controllable (z-spanning), and integrates seamlessly with the simulated-annealing reconstructions, making it robust and efficient for through-plane analysis (Figure 2b).

The 3D flood-fill algorithm (Figure 2a) provides a voxel-wise identification of the z-spanning cluster through a recursive neighbor search. Starting from the top-boundary voxels, the algorithm propagates through all connected voxels using a 26-neighbor criterion, ensuring that diagonal and edge contacts are included in the connectivity analysis. The propagation continues until no new voxels can be reached. If the connected region intersects the bottom boundary, the corresponding set of voxels is defined as the system-spanning (z-spanning) cluster, indicating a through-plane transport pathway. We then quantify the extent of long-range connectivity using a finite-size estimator of the infinite-cluster fraction, computed as the fraction of phase voxels that belong to the z-spanning cluster (denoted here as $P_{\infty }$ for brevity). This metric goes beyond a binary percolation test by measuring how much of the phase participates in the spanning network. We note that template-based microstructures (periodic lattices/scaffolds) can introduce geometric bias because predicted transport may depend strongly on arbitrary template parameters (e.g., pore shape, strut thickness, and unit-cell choice). In contrast, the percolation descriptor $P_{\infty ,z}(L)$ provides a consistent, architecture-agnostic measure of long-range connectivity across morphology families, without prescribing a single canonical geometry.

### 2.4. Connectivity Quality Metrics

While the percolating fraction $P_{\infty }$ indicates whether a through-plane path exists, the quality of that path controls the effective response. We therefore quantified four backbone-level metrics on the z-spanning cluster: (i) backbone fraction $f_{bb}$, defined as the fraction of percolating voxels that remain after pruning dead-ends; (ii) a bottleneck index B, taken as the 10th percentile of the cluster’s cross-sectional area along z, normalized by the median; (iii) through-plane tortuosity $\tau_{z}$, computed as the mean geodesic length divided by the sample thickness; and (iv) alignment $A_{z}=\langle cos\;\theta \rangle$, the average cosine between local backbone segments and the *z*-axis [28,46,47,48,49,50]. Intuitively, higher $f_{bb}$, larger B, lower $\tau_{z}$, and higher $A_{z}$ indicate fewer cul-de-sacs, fewer constrictions, straighter channels, and better directional alignment features that improve $\sigma_{\mathrm{eff}}$ even at fixed $P_{\infty }$.

For clarity, the main connectivity descriptors used in this work are as follows:

$P_{\infty }$: spanning-cluster fraction of the target phase (finite-size estimator), defined as the fraction of phase voxels belonging to the z-spanning connected component, $\left(|\Omega_{span}|/|\Omega_{\mathrm{phase}}|\right)$.

$f_{bb}$: backbone fraction, share of phase voxels that belong to the current-carrying backbone after pruning dead ends.

B: bottleneck index, a measure of the narrowest constrictions along the spanning backbone; higher B means fewer chokes.

$\tau_{z}$: through-plane tortuosity (larger values indicate more winding paths).

$A_{z}$: alignment factor toward z; larger values indicate stronger through-plane orientation.

Transport proxies.

$\sigma_{eff}$: effective electrical conductivity (component z when specified); $\sigma_{bulk}$: bulk reference.

$D_{eff}$: effective diffusivity (defined analogously on the pore phase).

${ASR}_{e}$: electronic area-specific resistance of the conducting path (including contacts, when applicable).

$R_{O_{2}}$: oxygen-transport resistance across GDL/CL (mass-transfer proxy).

$\left\langle J_{z}\right\rangle$: volume mean of the through-plane flux $J_{z}$ on the percolating domain.

### 2.5. Transport Simulations

The effective electrical conductivity of reconstructed porous electrodes is computed by lifting a validated 2D finite-volume framework to 3D and solving the transport problem on the z-spanning connected component detected via opposite-face connectivity (26-neighbor criterion). This preserves voxel-wise conservation, prevents spurious contributions from disconnected regions, and enables through-plane anisotropy analysis. The formulation is extended to 3D and restricted to the z-spanning cluster prior to solving Equation (5) subject to the boundary conditions in Equation (7), capturing 3D connectivity and tortuosity and aligning computations with percolation-based criteria commonly used in electrode analyses.

#### 2.5.1. Governing Problem and Boundary Conditions

Let $\Omega_{p}\subset R^{3}$ denote the percolating computational domain extracted from the reconstructed binary microstructure (150×150×150 voxels). In this work, $\Omega_{p}$ is defined as the system-spanning (z-spanning) connected component of the phase of interest identified in Section 2.4, i.e., $\Omega_{p}\equiv \Omega_{\mathrm{span},z}$. The steady electric potential $\phi \left(r\right)$ satisfies

(5) $$\nabla \cdot \left(\sigma \left(r\right)\nabla \phi \left(r\right)\right)=0\;in\;\Omega_{p}$$

The local conductivity is

(6) $$\sigma \left(r\right)=\left\{\begin{array}{cc} \sigma_{S}, & \mathrm{conducting}\;\mathrm{phase},\\ 0, & \mathrm{non-conducting}\;\mathrm{phase} \end{array}\right.$$

A prescribed potential difference is applied between the two opposite faces normal to z (e.g., ϕ=0 at z=0 and ϕ=Δϕ at $z=L_{z}$). The four lateral faces are treated as electrically insulating, such that no normal current crosses them:

(7) $$n\cdot \left(\sigma \nabla \phi \right)=0$$

where n is the outward unit normal. These boundary conditions enforce a through-plane potential drop while preventing lateral leakage currents, yielding $\sigma_{eff,zz}$ consistent with a unidirectional through-plane conduction test. Unit voxel spacing is assumed (Δx=Δy=Δz=1), so $L_{x}=L_{y}=L_{z}=150$ in voxel units. If no z-spanning connected component exists, the sample is classified as non-percolating in the through-plane direction and the transport solve is skipped (equivalently, $\sigma_{eff,zz}=0$).

#### 2.5.2. Numerical Scheme (Finite Volume Method)

A control-volume (FVM) discretization enforces local face-flux balance in each voxel

(8) $$\sum_{f\in \{E,W,N,S,T,B\}}\left(-\sigma_{f}\nabla \Delta \phi \cdot n_{f}\right)A_{f}=0,$$

where $\sigma_{f}$ denotes the face-interpolated conductivity, $A_{f}$ the face area (unity in voxel units), and $n_{f}$ the outward unit normal. The resulting symmetric positive-definite system is iteratively solved to a tight residual tolerance. This conservation framework matches the methodology previously employed to compute ETC with FVM in electrode in [51,52].

#### 2.5.3. Effective Properties and Normalization

For a given MEA layer (GDL or CL), the microstructure derived through-plane effective conductivity $\sigma_{eff,zz}$ provides a direct bridge to the layer’s ohmic behavior. Combining $\sigma_{eff,zz}$ with the layer thickness $L_{z}$ yields an area-specific resistance $\mathrm{ASR}=L_{z}/\sigma_{eff,zz}$ (in $\Omega \cdot {\mathrm{cm}}^{2}$ after unit conversion). Under an operating current density j, the corresponding voltage drop across the layer is $\Delta V_{\mathrm{layer}}=j\mathrm{ASR}$. In practice, the GDL contribution is dominated by the electronic network of the carbon matrix, whereas in the catalyst layer both the electronic (carbon) and protonic (ionomer) pathways matter and can be reported as separate effective conductivities. Summing the ASR of each layer, together with the membrane and interfacial contact terms, gives the MEA ohmic loss used in polarization curve analysis. This establishes a clear micro-to-macro link: the voxel scale solution furnishes the layer parameters needed by device scale models without additional fitting.

From the converged field,

(9) J(r)=−σ(r)∇ϕ(r).

The effective current $I_{eff}$(A) through the outlet plane $\Gamma_{z=L_{z}}$ is computed as

(10) $$I_{eff}=\int_{\Gamma_{z=L_{z}}}J\cdot n\;dA,$$

The through-plane effective conductivity is then

(11) $$\sigma_{eff,zz}=\frac{I_{eff}L_{z}}{A\Delta \phi },$$

where the cross-sectional area orthogonal to z is

(12) $$A=L_{x}L_{y}.$$

For cross-structure comparison, the normalized efficiency is reported as

(13) $$\eta =\frac{\sigma_{\mathrm{eff},zz}}{\sigma_{M}},$$

where J(r) is the local current density (${A\cdot m}^{-2}$), ϕ(r) is the electric potential (V), ∇ϕ is the potential gradient (${V\cdot m}^{-1}$), σ(r) is the local conductivity (${S\cdot m}^{-1}$), n is the outward unit normal, and dA is the surface element ($m^{2}$). The effective current $I_{eff}$ is the total current crossing the outlet plane (A). The domain lengths along the x, y, and z directions are $L_{x}$, $L_{y}$, and $L_{z}$(m), respectively; $L_{z}$ is the layer thickness (m). Δϕ is the imposed potential difference between the z-faces (V). $\sigma_{eff,zz}$ is the through-plane effective conductivity (${S\cdot m}^{-1}$), and $\eta =\sigma_{eff,zz}/\sigma_{M}$ is the dimensionless normalized efficiency, with $\sigma_{M}$ denoting the intrinsic (dense, non-porous) conductivity of the conducting phase (${S\cdot m}^{-1}$).

#### 2.5.4. Workflow and Implementation

Starting from a 3D binary voxel grid (1 = conducting phase, 0 = insulating phase), we first identify the system-spanning (z-spanning) connected component using a 26-neighbor connectivity criterion and define the computational domain as $\Omega_{p}\equiv \Omega_{\mathrm{span},z}$. If no z-spanning component exists, the sample is classified as non-percolating in the through-plane direction, $\sigma_{eff,zz}=0$, and the transport solve is skipped. On $\Omega_{p}$, the local conductivity field σ(r) is assigned according to Equation (6). The potential field ϕ is then obtained by solving Equation (5) subject to the boundary conditions in Equation (7), using the FVM flux-balance discretization in Equation (8), which ensures voxel-wise conservation and prevents disconnected clusters from contributing to through-plane transport. A prescribed potential drop is applied between the two opposite faces normal to z (e.g., $\phi =V_{0}$ at z=0 and ϕ=0 at $z=L_{z}$), while the four lateral faces are treated as electrically insulating, n·(σ∇ϕ)=0. The steady potential is obtained by solving the voxel-based conservation form of the conduction problem,

(14) $$-\nabla \cdot \left(\sigma \nabla \phi \right)=0\;\mathrm{in}\;\Omega_{p},$$

using a finite-volume discretization. From the converged field, the total current $I_{tot}$ through a plane normal to z is computed and used to evaluate the through-plane effective conductivity,

(15) $$\sigma_{eff,zz}=\frac{I_{tot}L_{z}}{A\;\Delta \phi },A=L_{x}L_{y},$$

and the normalized performance metric η is reported as defined in Equation (13). Optionally, repeating the same procedure with the imposed potential drop along x and y yields the diagonal entries of $K_{eff}$.

The connectivity screening (z-spanning connected-component detection) and the voxel-based finite-volume conduction solver were implemented i for efficient execution and transparent control of numerical settings. Post-processing, including parsing solver outputs, computing derived metrics ($\sigma_{eff,zz}$, η, and connectivity descriptors), aggregating ensemble statistics across realizations, and generating figures. Solver verification and mesh convergence are reported in Table 2 using an analytical two-layer benchmark.

#### 2.5.5. Correlation Model and Fitting Procedure

To relate connectivity descriptors to effective response, we considered the following phenomenological model:

(16) $$\frac{\sigma_{eff}}{\sigma_{bulk}}=C\;P_{\infty }^{\beta }B^{\delta }\tau_{z}^{-\gamma }A_{z}^{\eta }.$$

where $P_{\infty }$ is the spanning-cluster fraction (finite-size estimator of long-range connectivity), B the bottleneck index, $\tau_{z}$ the through-plane tortuosity, and $A_{z}$ the alignment; C, β, δ, γ, η are fitted parameters. We estimated parameters by nonlinear least squares on the percolating samples, with variables standardized to zero mean and unit variance before fitting. Uncertainty was assessed via bootstrap resampling, reporting 95% confidence intervals. Model diagnostics included residual analysis and variance inflation checks. No result interpretation is provided in this section; parameter estimates and trends are reported in Section 3.5 and discussed in Section 4.

### 2.6. Alternative Approaches and Perspectives

Recent advances have introduced data-driven and imaging-based methods for percolation assessment in porous media. Deep learning architectures—most commonly convolutional neural networks (CNNs), 3D U-Nets, and, more recently, graph neural networks (GNNs)—have been trained to infer percolating pathways or connectivity-related labels directly from 3D microstructural images (e.g., micro-CT or synchrotron tomography). These approaches can be attractive for rapid screening once trained, particularly in large parameter sweeps or when segmentation and connectivity labeling must be performed repeatedly. For example, De Beaufort et al. quantified electrode connectivity in PEMFCs using synchrotron X-ray tomography combined with machine-learning-based segmentation [53], and related data-driven strategies have been used to predict transport-relevant metrics in 3D heterogeneous structures [45].

However, percolation in voxelized media can also be solved deterministically by graph-based algorithms such as flood-fill (BFS/DFS) or union-find on the voxel adjacency graph. These methods are exact on the discretized geometry, require no training data, and provide fully interpretable labels of the system-spanning cluster with linear-time complexity O(N) in the number of voxels. In contrast, ML predictors typically deliver probabilistic outputs and may suffer from dataset shift when imaging conditions, materials, or morphology families differ from the training distribution; they also require curated labeled datasets and introduce additional hyperparameters and model-selection choices.

In the present study, we therefore employ a deterministic flood-fill approach to guarantee one-to-one voxel-level correspondence between geometric connectivity and the transport pathways used in the subsequent conductivity calculations, ensuring consistency and reproducibility across all reconstructions. At the same time, motivated by the potential throughput advantages of ML surrogates, we have initiated preliminary work toward CNN/GNN-based predictors trained on flood-fill ground-truth labels for rapid screening of large ensembles; such surrogates are intended as complementary accelerators, while deterministic connectivity remains the ground-truth engine for verification and reporting.

### 2.7. Use of GenAI and AI-Assisted Tools

During manuscript preparation (accessed November 2025), the authors used OpenAI GPT-5.1 Thinking solely for language polishing and terminology standardization. No data, analyses, equations, figures, or scientific conclusions were generated by AI. All AI-assisted text was reviewed and edited by the authors, who take full responsibility for the content. All figures, numerical results, and analyses were produced by the authors using their own datasets and codes, and no AI system generated any scientific results or images reported in the manuscript.

## 3. Numerical Configuration and Results

This section examines how three-dimensional connectivity governs effective transport in porous electrodes of membrane electrode assemblies (MEAs) using statistically reconstructed microstructures. Methods, algorithms, and solver formulations are detailed in Section 2; here, we focus on the computational setup, validation outcomes, connectivity metrics, and transport results.

### 3.1. Simulation Setup

Solver verification was carried out using a simple analytical two-layer benchmark (two equal-thickness layers in series along z, with $\sigma_{1}=1$ and $\sigma_{2}=10$), for which $\sigma_{eff}^{ana}=1.81818$. Across $100^{3}$, $150^{3}$, and $200^{3}$ grids, the computed $\sigma_{eff}$ stays within <0.55% of the analytical value and shows negligible variation (<0.01%) between the two finest meshes (Table 2). Overall, Table 2 confirms mesh convergence for the layered benchmark and provides visible support that the boundary-condition and discretization setup used for through-plane transport is numerically robust. Here, the analytical reference is the series (harmonic-mean) effective conductivity of two equal-thickness layers stacked along z:

(17) $$\sigma_{eff}^{ana}=\frac{2}{\left(\frac{1}{\sigma_{1}}\right)+\left(\frac{1}{\sigma_{2}}\right)},\;\sigma_{1}=1,\sigma_{2}=10$$

**Figure 3:**
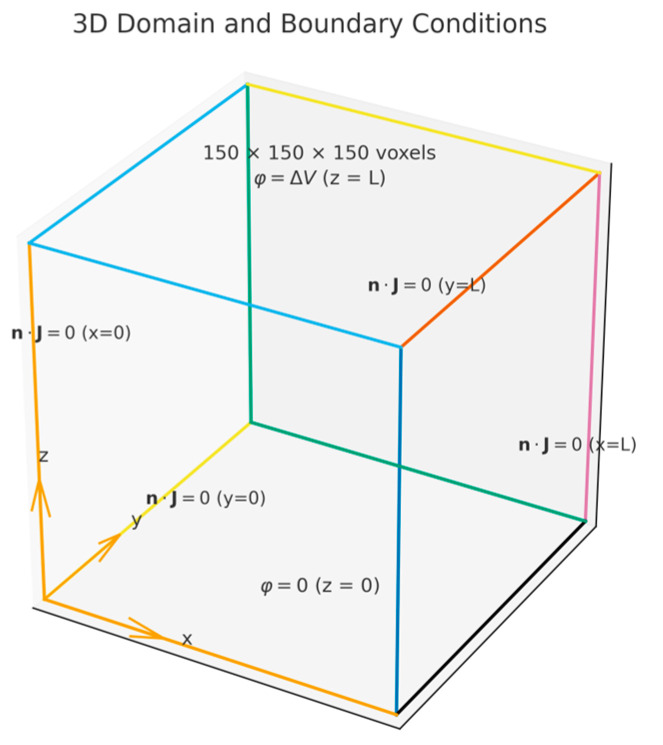
3D computational domain and boundary conditions used for through-plane effective conductivity simulations. Colors are used only to visually distinguish the coordinate directions/edges for clarity and do not represent additional boundary-condition information.

Accordingly, representative SEM micrographs were binarized and resampled to 150×150×150 voxel domains (Δx=Δy=Δz), using a fixed encoding (1 = solid, 0 = pore). Periodic boundaries were not used to preserve realistic through-plane connectivity, and the domain was oriented such that the *z*-axis represents the through-thickness transport direction relevant in MEAs (see Figure 3).

Three-dimensional volumes were reconstructed by simulated annealing. For each morphology family, we generated an ensemble of independent 3D reconstructions using identical simulated-annealing schedules and different random seeds. Reconstructions were retained only if they satisfied the descriptor-mismatch tolerance and through-plane percolation screening; for consistency, we report results using *N* = 4 accepted realizations per morphology. Unless otherwise stated, reported metrics ($P_{\infty }$ and $\sigma_{\mathrm{eff}}$) are computed over these realizations and reported as mean ± SD. For visualization, we selected the realization whose ($P_{\infty }$, $\sigma_{\mathrm{eff}}$) is closest to the morphology-specific ensemble median. This fixed sample size was used to enable a balanced comparison across morphology families under identical computational settings.

**Figure 4:**
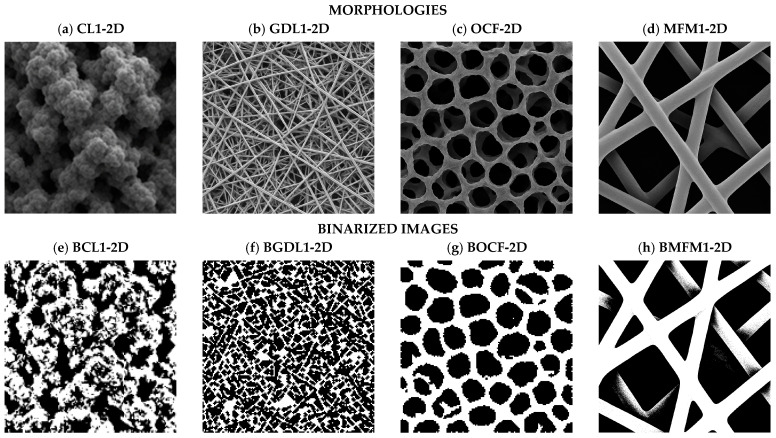
Synthetic SEM-like morphologies (2D) and their binarized 2D masks. Top row (**a**–**d**): 2D SEM-like exemplars for four morphology families (CL1, GDL1, OCF, and MFM1). Bottom row (**e**–**h**): corresponding 2D binarized masks with a fixed encoding (white = conductor, black = pore). This naming (2D/2D binarized) is used throughout the paper to distinguish image type from the 3D reconstructed volumes. We use the same identifiers to denote morphology families (CL1, GDL1, OCF, and MFM1). Here, “2D” refers to synthetic SEM-like images, “2D (binarized)” to their binary masks, and “3D” to reconstructed binary volumes (150 × 150 × 150 voxels). Unless stated otherwise, white = conductor and black = pore.

Before transport calculations, each volume was screened for through-plane (z-spanning) percolation using a 26-neighbor flood-fill applied to the phase of interest (solid for electronic conduction, pore for ionic scenarios) without altering the binary encoding. Only geometries with continuous top-to-bottom connectivity in the relevant phase proceeded with transport analysis. Boundary conditions for the transport simulations are summarized in Figure 3: fixed potentials on the two z-faces and no-flux on the four lateral faces, which drives through-plane transport without lateral leakage. Computations were performed in C (compiled with MinGW.org GCC 6.3.0-1), MATLAB R2018a (v9.4.0.813654), and Python 3.12.10 (NumPy 2.3.4, SciPy 1.16.3, Matplotlib 3.10.7). Post-processing was vectorized to compute microstructural descriptors, generate percolation masks, and integrate fluxes; solver tolerances and stopping criteria were kept fixed across all runs. Figure 4 summarizes the morphologies used for reconstruction and transport analyses. Figure 4a–d show synthetic SEM-like exemplars of CL (granular carbon-black aggregates), GDL (fibrous), OCF (open-cell foam), and MFM (micro-fibrous non-woven). Figure 4e–h display the corresponding binarized masks (BCL1, BGDL1, BOCF, and BMFM), following the fixed encoding 1 = solid (white) and 0 = pore (black). These masks preserve the salient features required by the statistical descriptors and serve as inputs for percolation screening and transport simulations.

### 3.2. Validation of the Reconstruction

Each reconstructed binary volume (150 × 150 × 150 voxels) was validated against its 2D statistical targets derived from the corresponding SEM exemplars (CL1-2D, GDL1-2D, OCF-2D, and MFM1-2D). Three descriptors were used as follows: the two-point correlation $S_{2}(r)$, the lineal-path function $L_{p}(r)$, and the pore-size function F(Ω,r). For every volume, descriptor profiles were computed along the three principal directions (x,y,z) to assess isotropy or directional bias. Reconstructions were accepted only when the aggregated normalized mismatch satisfied the preset tolerance, mean-squared error $\leq 10^{-6}$. Formal definitions, normalization, and the aggregation rule are given in Section 2. Throughout, the fixed encoding is white = solid (conductor) and black = pore.

#### 3.2.1. Statistical Targets and Acceptance Criterion

For each morphology family, target curves for $S_{2}$, $L_{p}$, and F(Ω,r) were computed from the 2D exemplars and used to guide reconstruction. The directional profiles along x, y, and z were compared to the corresponding volume averages from the reconstructed 3D masks. The directional errors were normalized by the target energies and then aggregated across descriptors and directions to produce a single acceptance metric. A volume was retained for downstream analysis only if the resulting normalized MSE met the tolerance $\leq 10^{-6}$. This threshold ensures descriptor-level fidelity that is tight relative to the morphological variability investigated in Section 3.3 and Section 3.4.

#### 3.2.2. Morphology

The dataset spans four archetypes: CL1 (granular, catalyst-layer-like agglomerates), GDL1 (random fibrous gas-diffusion layer), OCF (open-cell foam, used as a morphological control), and MFM1 (micro-fibrous, non-woven). Descriptor trends are consistent with visual appearance. OCF shows near-isotropy with rounded pores and smooth struts; CL1 is also close to isotropic but with broader pore-size distribution due to hierarchical roughness. Fibrous architectures (GDL1 and MFM1) exhibit strong in-plane correlation in $S_{2}$ and $L_{p}$, thinner through-plane backbones, and narrower F(Ω,r) peaks that reflect more defined length scales. These traits anticipate robust solid-phase percolation in all cases, while pore-phase percolation depends on window necks in OCF, inter-fiber spacing in GDL1/MFM1, and channel continuity in fabrics with woven-like features.

#### 3.2.3. Visual Inspection and Link to Downstream Analyses

**Figure 5:**
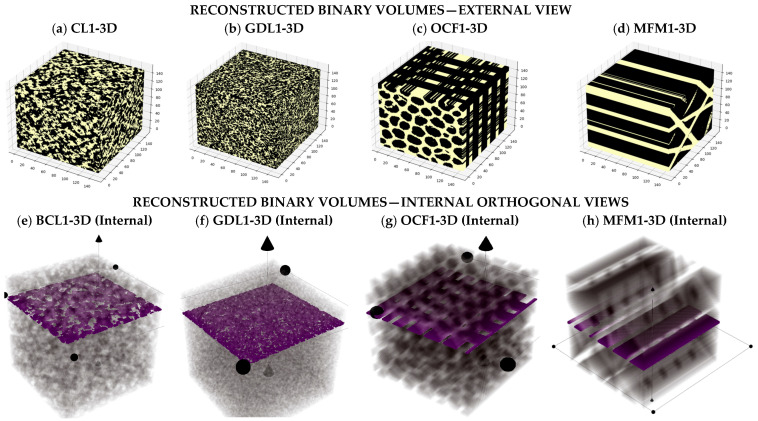
Reconstructed binary volumes (3D): external and internal views. Top row (**a**–**d**): external renderings of CL1-3D, GDL1-3D, OCF1-3D, and MFM1-3D (150 × 150 × 150 voxels), where the conducting (solid) phase is shown in yellow (background omitted for clarity). Bottom row (**e**–**h**): internal orthogonal views of the same volumes. Magenta planes mark representative section cuts used for subsequent layer-wise analyses of the conducting fraction per layer $f_{z}$ and the normalized through-plane flux $J_{z}$. The through-plane direction z is indicated in each panel by the axis/arrow marker.

Figure 5 illustrates the accepted 3D masks. The top row shows external renderings of the reconstructed binary volumes for CL1-3D, GDL1-3D, OCF-3D, and MFM1-3D (Figure 5a–d). The bottom row shows internal orthogonal views of the same volumes (Figure 5e–h). The magenta planes indicate example sections used later for layer-wise analysis of the conducting fraction per layer $f_{Z}$ and of the normalized through-plane flux $J_{Z}$. The through-plane direction Z is indicated in each panel. All four volumes pass the acceptance criterion based on the aggregated normalized MSE.

### 3.3. Connectivity and Percolation Analysis

The phase and sign convention used in this analysis is shown as follows. White denotes the solid (conducting) phase, and black denotes the pore phase. Connectivity is evaluated on the phase of interest. The algorithm and its classification are described below. We apply a 26-neighbor flood-fill (faces, edges, and corners) seeded with all voxels of the target phase on the inlet plane z=0. A volume is classified as top-to-bottom percolating if the visited cluster also touches the outlet plane at $z=L_{z}$. Using 26-connectivity avoids artificial disconnections of diagonally touching voxels and typically yields an upper bound relative to 6- or 18-neighbor definitions. For each percolating volume, we compute the percolating fraction of the target phase, $P_{\infty }$, defined as

(18) $$P_{\infty }=\frac{\left|\Omega_{\infty }\right|}{\left|\Omega_{phase}\right|}$$

where $\Omega_{\infty }$ is the set of voxels belonging to the system-spanning cluster and $\Omega_{\mathrm{phase}}$ is the full set of voxels of the analyzed phase. Here, $P_{\infty }$ represents the fraction of phase voxels that actively participate in a continuous through-plane (z-spanning) backbone; values approaching unity indicate that most of the phase network contributes to the through-thickness connected skeleton. Non-percolating volumes are retained for completeness but excluded from transport simulations and reported with $\sigma_{eff,zz}=0$ (or $D_{eff,zz}=0$ for diffusivity), because they cannot sustain through-plane transport under the imposed boundary conditions. In this work, we report (i) the fraction of z-spanning volumes for each morphology and (ii) the distribution of $\phi_{p}$ among percolating cases. Case-by-case and aggregated results are summarized in Table 3 and Table 4. Binary masks are analyzed as generated, without any morphological editing.

Across the four exemplars, all volumes percolate top to bottom, with $P_{\infty }$ ranging from 0.901 to 0.999. GDL1 and OCF exhibit near-unity $P_{\infty }$, consistent with a continuous backbone and open-cell topology, respectively. CL1 also shows a near-unity $P_{\infty }$, indicating a consolidated solid skeleton. In contrast, the micro-fibrous non-woven (MFM1) achieves percolation but with a lower $P_{\infty }$ = 0.901, suggesting bottlenecks and dangling ends within the current-carrying network. As detailed later, $P_{\infty }$ acts as a first-order predictor of $\sigma_{eff,zz}$, while path-quality descriptors such as the bottleneck index B, tortuosity $\tau_{z}$ alignment $A_{z}$, and backbone content $f_{bb}$ modulate the magnitude at comparable $P_{\infty }$. Using 26-connectivity avoids artificial disconnections between diagonally touching voxels and provides an upper bound relative to 6- or 18-neighbor criteria; binary masks were analyzed as generated, without morphological editing.

### 3.4. Effective Transport Coefficients

Transport simulations are restricted to through-plane (z-direction) domains of the solid conducting phase. A volume is spanning only if a 26-neighbor flood-fill started at z = 0 reaches z = L. Non-spanning volumes are kept for completeness, reported as $\sigma_{eff}$ = 0 under the stated boundary conditions, and excluded from ensemble aggregates. Encoding is fixed throughout: white = solid (conducting), black = pore. In all simulations, the microstructures are aligned with z as the through-thickness axis, reconstructed and validated as described in Section 2 and Section 3 on a 150 × 150 × 150 binary grid (1 = solid, 0 = pore), and steady-state transport is solved with a finite-volume method (FVM) using fixed potentials on the two faces normal to z to drive through-plane transport and no-flux boundary conditions on the four lateral faces (x and y). Results are reported as $\sigma_{eff}$/$\sigma_{bulk}$.

**Figure 6:**
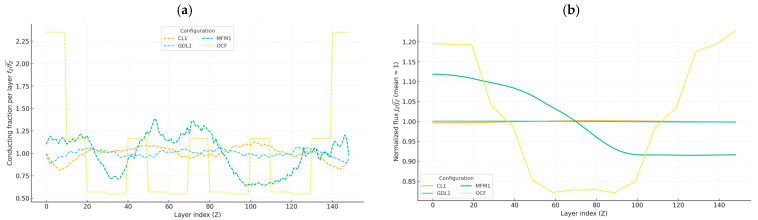
Structure versus layer-wise flux (white phase = conductor). (**a**) Layer-wise conducting fraction $f_{z}$, normalized by the volume mean $\left\langle f_{z}\right\rangle$, along the thickness Z for CL1, GDL1, OCF, and MFM1. (**b**) Layer-averaged through-plane flux $J_{z}$, normalized by the volume mean $\left\langle J_{z}\right\rangle$ (so the mean equals 1), along Z for the same configurations. Taken together, (**a**,**b**) reveal the expected inverse trend: layers with larger connected conducting cross-section tend to carry above-average flux; deviations indicate connectivity effects such as dead ends, constrictions, or high tortuosity. OCF shows step-like plateaus from its channel/barrier pattern; MFM1 exhibits a laminated gradient; CL1 and GDL1 are nearly flat, consistent with more homogeneous connectivity.

For each configuration we summarize, as mean ± SD over replicate reconstructions: (i) the fraction of volumes that span in z and (ii) the percolating fraction of the conducting phase, $\phi_{p}$, measured on the spanning cluster. The workflow enforces three basic convergence checks: (i) a residual tolerance consistent with Section 2, (ii) inlet–outlet flux balance to ensure conservation, and (iii) ΔV-scaling invariance, so that the computed $\sigma_{\mathrm{eff}}$ is independent of the imposed potential difference. When performed, mesh-refinement or patch tests (e.g., increasing the resolution from 150^3^ to a finer grid or refining selected regions) confirm that discretization errors are negligible compared with the variability induced by microstructural differences. Figure 6 couples structure and transport along z by overlaying the normalized layer-wise conducting fraction, $f_{z}/\langle f_{z}\rangle$, and conducting-phase flux, $J_{z}/\langle J_{z}\rangle$. Inverse trends indicate simple redistribution with available cross-section, while deviations and the 3D rendering of the percolating cluster reveal connectivity constraints in the current-carrying backbone.

For each configuration, the normalized through-plane conductivity $\sigma_{eff}/\sigma_{bulk}$ is reported together with the resulting current magnitude $|I_{eff}|$ under the boundary conditions in Section 2 (fixed potential drop across z, no-flux on lateral faces). In-plane components (x,y) are shown when available to indicate anisotropy. When raw outputs include sign due to field orientation, values are reported here as magnitudes. We also provide Eff% = $100\times \sigma_{eff}/\sigma_{bulk}$ for readability.

CL1 (granular).

Through-plane: $\sigma_{eff}/\sigma_{bulk}=0.306$ (Eff% = 30.6); $|I_{eff}|=4.59\times 10^{4}$.

In-plane: x≈0.307, y≈0.310 (low anisotropy).

GDL1 (random fibrous GDL).

Through-plane: $\sigma_{eff}/\sigma_{bulk}=0.303$ (Eff% = 30.3); $|I_{eff}|=4.55\times 10^{4}$.

In-plane: x≈0.302, y≈0.303 (low anisotropy).

OCF (open-cell foam, control).

Through-plane: $\sigma_{eff}/\sigma_{bulk}=0.706$ (Eff% = 70.6); $|I_{eff}|=1.06\times 10^{5}$.

In-plane: x≈0.650, y≈0.666 (low anisotropy, about 8 to 9%).

MFM1 (micro-fibrous, non-woven).

Through-plane: $\sigma_{eff}/\sigma_{bulk}=0.134$ (Eff% = 13.4); $|I_{eff}|=2.01\times 10^{4}$.

In-plane: x≈0.284, y≈0.101 (marked anisotropy, x≫y).

**Figure 7:**
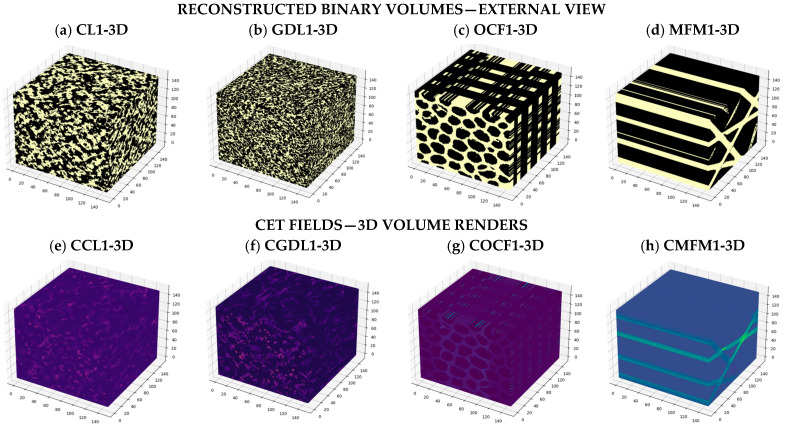
Reconstructed binary volumes and 3D current-density fields. Top row (**a**–**d**): external renderings of accepted binary volumes for CL1-3D, GDL1-3D, OCF1-3D, and MFM1-3D (150 × 150 × 150 voxels), where the conducting (solid) phase is shown in light yellow and the pore phase is omitted for clarity. Bottom row (**e**–**h**): steady-state CET fields obtained from the finite-volume solver under an imposed potential difference on the z-faces and no-flux boundary conditions on the lateral faces. Colors map the normalized current-density magnitude (brighter colors indicate higher current density). The through-plane direction (z) is indicated in each panel. Renders highlight the z-spanning skeleton and bottlenecks: OCF concentrates flux in vertical channels; MFM1 localizes it along inter-lamella bridges; CL1/GDL1 show more diffuse, isotropy-consistent patterns. All four volumes percolate through-plane; non-spanning cases would yield negligible current under identical boundary conditions.

Synthesis. Among these four configurations, the open-cell foam exhibits the largest normalized through-plane response; CL1 and GDL1 show similar mid-range values with low anisotropy, and MFM1 presents the lowest through-plane conductivity and strong in-plane anisotropy, consistent with preferential alignment of fibrous backbones. This ordering is consistent with increased continuity of the through-thickness skeleton in OCF and aligns with the structure–property analysis in Section 3.2 and Section 3.3. Representative 3D renderings and steady-state current-density fields are shown in Figure 7, highlighting morphology-dependent current localization patterns and the role of bottlenecks and backbone continuity under identical boundary conditions In layer-wise overlays, $J_{z}/\langle J_{z}\rangle$ redistributes toward well-connected regions; when additional area is poorly connected, the flux curve departs from a simple anti-correlation.

### 3.5. Structure–Transport Correlation

Across all datasets, through-plane transport displays a clear percolation-controlled signature. Samples without a spanning cluster exhibit negligible normalized conductivity $\sigma_{eff}/\sigma_{bulk}\approx 0$. The appearance of a through-plane cluster is accompanied by a sharp increase in response. Among percolating cases, $\sigma_{eff}/\sigma_{bulk}$ increases monotonically with the percolating fraction of the conducting phase $\left(\phi_{p}\right)$, with the steepest gains close to the onset. These trends are robust under simple resampling checks and remain visible after stratifying by morphology. Microstructural descriptors help explain why samples with comparable $\phi_{p}$ can still differ in transport. Two-point statistics indicate that microstructures with more persistent correlation in the through-plane direction tend to percolate at lower overall solid fraction and to allocate a larger share of the solid to the connected skeleton. Isotropic granular textures generally perform at or above the global trend for a given $\phi_{p}$, which suggests more continuous and well distributed pathways. Fibrous or woven architecture often falls below the trend unless deliberate vertical bridging is present. Outliers are linked to thread-like contacts, narrow constrictions, or pathway segments predominantly aligned in plane.

### 3.6. Sensitive Analysis

The conducting-phase fraction in the k-th layer along *z* is denoted ${\;f}_{z}(k)$. The normalized profile is defined as ${\tilde{f}}_{z}(k)={\;f}_{z}(k)/{\bar{f}}_{z}$ where ${\bar{f}}_{z}$ is the mean value of ${\;f}_{z}(k)$ across all layers (thus mean($\left({\tilde{f}}_{z}\right)=1$). The bottleneck index is defined as $L_{z}=1/{min}_{k}\left\{{\tilde{f}}_{z}(k)\right\}$ and the uniformity score as $\varepsilon_{z}=1-CV\left({\tilde{f}}_{z}\right)$, where $CV\left({\tilde{f}}_{z}\right)=std\left({\tilde{f}}_{z}\right)/mean\left({\tilde{f}}_{z}\right).$ The resulting layer-wise descriptors and the normalized through-plane conductivity for each morphology are summarized in Table 5.

Pearson correlation coefficients (r) computed across the four studied morphologies (N = 4) are shown in Table 6.

## 4. Discussion

The percolation-aware workflow developed in this study provides a practical screening logic for real porous architectures: it first verifies the existence of a through-plane system-spanning pathway, and then rationalizes performance differences among percolating samples through pathway-quality descriptors (constrictivity/bottlenecks, tortuosity, and anisotropy). This perspective is consistent with prior microstructure–property studies in electrochemical porous layers reporting strong sensitivity of effective transport to constrictivity/tortuosity and compression-driven anisotropy.The present results highlight a practical design asymmetry between gas diffusion layers and catalyst layers in MEAs. Gas diffusion layers can deliver useful through-plane connectivity at moderate solid fractions, provided that processing promotes vertical bridging and that operational compression is leveraged to improve contact between fibers. In contrast, granular catalyst layers generally require higher solid fractions, of the order of 0.7 in our dataset, to consolidate a continuous electronic skeleton across thickness. For GDLs, the priority is not simply to add solid, but to engineer through-plane bridges and to limit excessive in-plane alignment that penalizes the measured direction. For CLs, increasing the solid fraction toward this range raises the likelihood of a robust spanning skeleton, but such gains must be balanced against device-level constraints and potential trade-offs in oxygen transport and water management.

These design trends are consistent with the image-based 3D workflow summarized in Figure 1, which reconstructs statistically consistent volumes from SEM input, identifies the z-spanning cluster and quantifies its contribution to macroscopic response. By applying this workflow to four archetypal architectures, we show that even when all samples percolate through the thickness, their normalized effective conductivity $\sigma_{eff}$/$\sigma_{bulk}$ differs markedly because of microstructural details of the percolating skeleton. Open-cell foams, with vertically coherent channels, exhibit the largest $\sigma_{eff}$/$\sigma_{bulk}$. Micro-fibrous non-woven supports underperform due to laminated constrictions and thread-like contacts, whereas granular CL and fibrous GDL textures fall in an intermediate range with nearly isotropic skeletons. Thus, the 3D SEM-based pipeline does not merely reconstruct morphology but provides a percolation-aware basis to compare contrasting MEA architectures on the same footing.

Importantly, $P_{\infty }$ is not a geometric template; rather, it is a percolation-based connectivity gatekeeper that generalizes across architectures and provides a consistent criterion for restricting transport calculations to the system-spanning domain in finite voxel samples. This distinction is particularly relevant for heterogeneous and multiphase electrode-like media, where idealized scaffold/periodic-template simplifications can obscure phase distribution, interfacial effects, and anisotropic pathways, and therefore bias effective-property predictions unless they are carefully calibrated against image-based or experimentally informed microstructures [16,32,35,54,55,56,57].

The link between percolation and transport also clarifies why morphologies with comparable percolating fraction $P_{\infty }$ can diverge in performance. At fixed $P_{\infty }$, the magnitude of $\sigma_{eff}$ is governed by path-quality descriptors. Higher bottleneck index B and alignment $A_{z}$, and lower through-plane tortuosity $\tau_{z}$, systematically improve both electronic conductivity and diffusive transport along z. Isotropic granular textures tend to allocate a larger share of solid to well-distributed paths and therefore perform at or above the global ($P_{\infty }$, $\sigma_{eff}$) trend. Fibrous or woven textures often require deliberate vertical bridging to avoid constrictions and thread-like contacts that limit through-plane response despite high $P_{\infty }$. This structure-level insight complements bulk metrics such as total solid fraction and helps identify processing levers that matter most for conductivity and oxygen transport. Several practical factors beyond the present scope may influence measured conductivity in real MEAs; these limitations and straightforward model extensions are summarized in Section 4.1. First, finite voxel size and segmentation thresholds can bias the detection of narrow connections; sensitivity analyses partly mitigate this risk, but experimental tomography at matched resolution would strengthen validation. Second, while the present study focuses on electronic transport in the conducting phase, the same workflow can be applied to diffusive transport in the pore phase, enabling cross-property trends between $\sigma_{eff}$ and $D_{eff}$ and a more integrated view of MEA performance. From an MEA perspective, $P_{\infty }$ marks the onset of non-negligible through-plane response. For samples with similar $P_{\infty }$, larger B and $A_{z}$ and smaller $\tau_{z}$ increase $\sigma_{eff}$ and $D_{eff}$; consequently, electronic area-specific resistance and oxygen-transport resistance decline in tandem. Conversely, skeletons rich in dead ends, that is, with low backbone fraction $f_{bb}$, divert volume away from the current-carrying backbones and correlate with higher electronic ASR and elevated qualitative flooding risk. In our set, granular CLs require higher solid fraction to secure a robust backbone at acceptable B and $\tau_{z}$, whereas fibrous GDLs benefit from compression-assisted alignment that increases $A_{z}$ without imposing severe constrictions. Overall, the descriptors that track percolation and pathway quality, namely $P_{\infty }$, $f_{bb}$, B, $\tau_{z}$, and $A_{z}$, provide a compact bridge between microstructure and MEA-level targets. They can serve as screening variables in a multi-objective optimization framework that balances conductivity and oxygen-transport goals with water-management risk, material usage, and processing constraints, thereby enabling rational formulation and manufacturing routes for high-performance MEAs.

Beyond initial performance, the electronic network in porous MEA layers can influence both the evolution and the partial recovery of voltage losses during lifetime operation. Recent PEMFC life-prediction studies explicitly include recovery mechanisms for reversible voltage loss, indicating that a fraction of the observed voltage decay can recover under suitable recovery conditions rather than being strictly permanent [58]. Because electrode-side ohmic polarization depends on the integrity and redundancy of the conducting skeleton, higher spanning connectivity $P_{\infty }$, together with robust backbone quality (higher backbone fraction $f_{bb}$, higher bottleneck robustness B, lower through-plane tortuosity $\tau_{z}$, and favorable alignment A), provides a microstructural rationale for reduced susceptibility to increases in ohmic resistance and for interpreting recovery phenomena in diagnostic or life-prediction models. In the present work, these descriptors are computed directly on the system-spanning domain, thereby offering quantitative structural priors to screen electrode architectures: networks with greater redundancy and weaker constrictions are expected to be less vulnerable to localized damage (e.g., fiber breakage/displacement) that can trigger abrupt losses in effective connectivity. This framing is especially relevant for fibrous layers (GDL-like media), where compression can modify interfacial contact resistance and induce microstructural damage or rearrangement that affects electronic pathways. While our framework is not a time-dependent degradation model, it establishes a measurable link between initial skeleton robustness and electrode-side ohmic behavior.

### 4.1. Limitations and Practical Factors

The current framework computes bulk conduction on fixed voxel geometries and therefore does not include contact resistance, humidity effects, mechanical compression, or operational microstructural evolution. These factors can substantially shift the experimentally measured conductivity; thus, the reported $\sigma_{eff}$ should be interpreted as the intrinsic connectivity-driven contribution of the reconstructed microstructure. Future extensions can incorporate (i) contact resistance via interfacial resistor networks or boundary resistance terms, (ii) humidity-dependent material parameters capturing changes in ionomer/water distribution, and (iii) compression through morphological compaction operators and/or contact-law models, as well as time-dependent microstructural updates to represent degradation.

### 4.2. Validation Outlook and Future Benchmarking

A direct experimental validation of $\sigma_{eff}$ is not included in the present study and would require a matched geometry–boundary-condition dataset: (i) 3D imaging of the tested electrode (e.g., X-ray micro-CT or FIB-SEM/serial sectioning), or statistically consistent reconstructions derived from microscopy; (ii) through-plane (or in-plane) electrical resistance/conductivity measurements under controlled compression and humidity with matched thickness and cross-sectional area; and (iii) separation of bulk conduction from interfacial/contact contributions. Such measurements would enable a one-to-one benchmark of the present voxel-based conduction solver and provide calibration parameters for extended models that include contact resistance (e.g., boundary-resistance terms or interfacial resistor networks). In this work, SEM micrographs are used as morphological input for reconstruction rather than as an experimental benchmark of $\sigma_{eff}$; therefore, experimental validation is identified as a priority direction for future work.

## 5. Conclusions

Through-plane transport in MEA electrodes is governed by three-dimensional percolation. The absence of a z-spanning connected component implies a negligible effective response. Once a continuous conducting skeleton appears, the normalized conductivity $\sigma_{eff}/\sigma_{bulk}$ increases monotonically with the percolating fraction $P_{\infty }$, establishing $P_{\infty }$ as a practical 3D connectivity criterion to determine whether a microstructure provides useful through-plane conduction paths. For a given $P_{\infty }$, the magnitude of $\sigma_{eff}$ is controlled by pathway-quality descriptors, including bottlenecks, tortuosity, alignment, and backbone content. Microstructures with higher bottleneck robustness (e.g., higher B), higher alignment $A_{z}$, and lower through-plane tortuosity $\tau_{z}$ systematically exhibit higher $\sigma_{eff}$ (and $D_{eff}$ where applicable), implying lower electronic ASR and reduced oxygen-transport resistance.

From a design perspective, GDLs can achieve useful connectivity at moderate solid fractions if processing promotes vertical bridges and leverages operational compression, whereas granular CLs generally require higher solid fractions to consolidate a robust spanning skeleton. The four archetypal architectures analyzed here—granular CL-like, fibrous GDL-like, open-cell foam, and micro-fibrous non-woven morphologies—occupy distinct regions in the $\left(P_{\infty },\sigma_{eff}/\sigma_{bulk}\right)$ space and differ in pathway-quality metrics, illustrating that the geometry of the percolating skeleton, rather than phase amount alone, controls performance. In both layers, increasing solid content must be balanced against device-level constraints and water-management trade-offs; therefore, improving backbone quality can be as important as increasing $P_{\infty }$.

Actionable optimization criteria (this study). The descriptors $P_{\infty }$, $f_{bb}$, B, $\tau_{z}$, and $A_{z}$ provide practical screening metrics for MEA optimization: (i) a connectivity gatekeeper requiring a nonzero z-spanning cluster ($P_{\infty }>0$); (ii) backbone and bottleneck quality favoring higher backbone participation ($f_{bb}$) and weaker constrictions (higher B); (iii) a transport-penalty criterion favoring lower through-plane tortuosity ($\tau_{z}$); and (iv) directional pathway support favoring higher through-plane alignment ($A_{z}$) to reinforce through-plane conduction paths. Combined with the proposed SEM-based 3D workflow, these descriptors enable microstructure-level screening within multi-objective design frameworks that balance conductivity and oxygen transport performance with water-management risk, material usage, and processing constraints.

An important application direction is to embed microstructure-derived skeleton descriptors (e.g., $P_{\infty }$ and bottleneck/alignment/tortuosity metrics) as structural priors in online PEMFC health estimation frameworks based on polarization-loss decomposition. In such approaches, activation/ohmic contributions can be decoupled and the evolution of the ohmic term can be tracked using high-frequency resistance (HFR) and related diagnostics [59]. In this way, skeleton descriptors could help interpret or constrain the electrode-side contribution to ohmic polarization (e.g., connectivity robustness and susceptibility to contact-network degradation), thereby linking microstructure screening/optimization with system-level diagnosis and lifetime management [60]. Beyond PEMFC MEAs, the same microstructure–connectivity perspective is relevant to other porous functional platforms, such as porous silicon multilayers used for optical/chemical sensing [61].

## Figures and Tables

**Table 1:** Simulated annealing (SA) configuration and reproducibility settings used for 3D microstructure reconstruction.

Parameter	Value/Definition
Reconstruction domain size	150 × 150 × 150 voxels (L = 150).
Phase representation	Binary voxels: solid = 1, pore = 0
Initialization (pre-volume)	Initial 3D configuration generated using pore-size–based constraints (maximum radius = 20) to satisfy the prescribed phase fraction; SA then refines the morphology by matching correlation descriptors.
Target descriptors	Two-point correlation $S_{2}$ (r) and lineal-path function $L_{p}$ (r), computed for both phases (solid and pore).
Number of lags	N_lags = ⌊L/2⌋ = 75 (for L = 150).
Objective function	Lag-averaged mean-squared mismatch between target (2D) and reconstructed (3D) descriptors aggregated over both phases: $S_{2}$ and $L_{p}$ (Equation (4)).
Initial temperature	$T_{0}=1\times 10^{-10}$.
Cooling schedule	Geometric: $T_{k+1}=\alpha T_{k}$
Cooling coefficient	α = 0.999999999 (fixed value).
Temperature update frequency	After each attempted trial move (one trial move per temperature update).
Move set	Randomly select one voxel in phase 1 and one voxel in phase 0 and swap labels (1 ↔ 0).
Acceptance rule	Metropolis: accept if ΔE ≤ 0; otherwise accept with probability exp(−ΔE/T).
Convergence tolerance	$\varepsilon =5\times 10^{-10}$(stop if objective error E < ε).
Minimum temperature	$T_{min}=T_{0}(0.1{)}^{20\lceil {log}_{2}(L)\rceil }$. For L=150: $\lceil {log}_{2}(150)\rceil =8$ → $T_{min}=10^{-166}$
Stopping criteria (implemented)	SA loop runs while $\left(T>T_{min}\right)$ AND (*E* > ε); stops when either ${T\leq T}_{min}$ or *E* ≤ ε.
Weighting factors (ϕ)	Equal weights for all mismatch terms: $\phi_{S2,solid}=\phi_{Lp,solid}=\phi_{S2,pore}=\phi_{Lp,pore}=1$.
Random-seed policy	Randomly select one voxel in phase 1 and one voxel in phase 0 and swap labels (1 ↔ 0), preserving the global phase fraction.
Output	Reconstructed 3D volume

**Table 2:** Layered benchmark: mesh convergence of the through-plane effective conductivity ($\sigma_{eff}$).

Mesh	$\sigma_{eff}$ (num)	$\sigma_{eff}$ (ana)	Error (%)
100^3^	1.82807474	1.81818182	0.5441
150^3^	1.82595908	1.81818182	0.4277
200^3^	1.82574987	1.81818182	0.4162

**Table 3:** Case-by-case percolation results.

Case ID	Morphology	Percolates (Top–Bottom)	*P*_∞_ (Solid)
CL1	CL (granular)	Yes	0.996
GDL1	GDL (fibrous)	Yes	0.999
MFM	Micro-fibrous non-woven	Yes	0.901
OCF	Open-cell foam (control)	Yes	0.997

**Table 4:** Aggregated percolation statistics by morphology. Values are reported as the mean across the accepted realizations (N = 4). $P_{\infty }$ denotes the percolating (top–bottom spanning) fraction of the phase of interest.

Morphology	N	Top–Bottom Percolation (%)	*P*_∞_ (Mean)	Notes
CL (granular)	4	4/4 (100.0%)	0.996	Hierarchical roughness; tortuous pores
GDL (fibrous)	4	4/4 (100.0%)	0.999	Solid backbone; directional channels
Micro-fibrous non-woven	4	4/4 (100.0%)	0.901	Non-woven network; bottlenecks
Open-cell foam (control)	4	4/4 (100.0%)	0.997	Near-isotropic; narrow length scale

**Table 5:** Layer-wise skeleton descriptors (through-plane, *z*-direction) and normalized through-plane conductivity for each morphology.

Morphology	σ_eff,zz_/σ_bulk_	ϕ*_z_* = *f_z_*	*L_z_*	*ε_z_*
CL1	0.306	0.420	1.194	0.933
GDL1	0.303	0.383	1.144	0.957
MFM1	0.134	0.315	1.041	0.961
OCF	0.706	0.186	3.319	0.776

**Table 6:** Pearson correlation matrix (compact) using independent layer-wise descriptors.

Pearson r	*σ_tp_*	ϕ*_z_*	*L_z_*
$\sigma_{tp}$	1.00	−0.72	0.96
ϕ*_z_*	−0.72	1.00	−0.88
$L_{z}$	0.96	−0.88	1.00

## Data Availability

The original contributions presented in this study are included in the article/Supplementary Material. Further inquiries can be directed to the corresponding author.

## Supplementary Materials

The following supporting information can be downloaded at: https://www.mdpi.com/article/10.3390/ma19050835/s1, Table S1 (literature benchmark for electrode-side resistances in PEMFC diffusion-media assemblies) [62,63,64,65].
