# Supplementary material for: Three-Dimensional Infinite Cluster Function as a Descriptor of Through-Plane Effective Conductivity in Porous Electrodes of Membrane Electrode Assemblies
Source: Materials (Basel). 2026 Feb 24;19(5):835. doi: 10.3390/ma19050835 (PMC12986540; doi:10.3390/ma19050835)
Supplement: Supplementary file 1 [file materials-19-00835-s001.zip › materials-4057605-supplementary.pdf]

Table S1. Literature benchmark for order-of-magnitude electrode-side resistances in PEMFC diffusion-media assemblies (bulk vs. contacts).

Quantity (representative PEMFC diffusion-media assembly)	Representative value	Implied/Notes
GDL through-plane electrical resistivity, ρzz	80 mΩ·cm (≈ 800 μΩ·m)	Torayca™ carbon paper TGP-H-060; thickness direction; representative value. [S1]
Representative GDL thickness, Lz	0.19 mm	Nominal thickness for TGP-H-060. [S1]
Bulk GDL electronic ASR, ASRbulk = ρzz Lz	≈ 1.52 mΩ·cm^2	Computed from 80 mΩ·cm × 0.019 cm. [S1]
Measured through-plane resistance (GDL with MPL, "GDL-30")	11.9 mΩ·cm^2	Reported experimental benchmark for through-plane resistance including effects beyond bulk. [S2]
BPP/GDL interfacial contact resistance (ICR)	Minimum 4.75 mΩ·cm^2	After compression break-in; strongly depends on pressure/roughness/coatings. [S3]
Contact resistances share of total I^2R losses (state-of-the-art PEMFC)	55%	After accounting for bulk resistances, contacts dominate total I^2R losses in that assembly [S3].
Clamping-pressure dependence (qualitative)	Higher pressure → lower ICR (trade-off)	Higher pressure increases real contact area but may over-compress GDL and increase flow resistance [S4].

Notes: Values are order-of-magnitude and depend on compression, MPL/coatings, and operating conditions. ρ_{zz}refers to bulk resistivity in the thickness direction (manufacturer unit mΩ·cm). $ASR_{bulk}$is computed from the reported $\rho_{zz}$and $L_z$values.

REFERENCES (Supplementary)

[S1] M. P. Chavhan, V. Slovak, G. Zelenkova, and D. Dominko, "Revisiting the Effect of Pyrolysis Temperature and Type of Activation on the Performance of Carbon Electrodes in an Electrochemical Capacitor," *Materials*, vol. 15, no. 7, Art. 2431, 2022, doi: **10.3390/ma15072431**.

[S2] V. M. Truong, N. B. Duong, C.-L. Wang, and H. Yang, "Effects of Cell Temperature and Reactant Humidification on Anion Exchange Membrane Fuel Cells," *Materials*, vol. 12, no. 13, Art. 2048, 2019, doi: **10.3390/ma12132048**.

[S3] C. J. Netwall, B. D. Gould, J. A. Rodgers, N. J. Nasello, and K. E. Swider-Lyons, "Decreasing contact resistance in proton-exchange membrane fuel cells with metal bipolar plates," *Journal of Power Sources*, vol. 227, pp. 137–144, 2013, doi: **10.1016/j.jpowsour.2012.11.012**.

[S4] X. Lai, D. Liu, L. Peng, and J. Ni, "A mechanical–electrical finite element method model for predicting contact resistance between bipolar plate and gas diffusion layer in PEM fuel cells," *Journal of Power Sources*, vol. 182, no. 1, pp. 153–159, 2008, doi: **10.1016/j.jpowsour.2008.03.069**.
